# Can Malawi afford to lose? The definitive cost of ‘brain drain’ and ‘brain waste’ in its essential medical laboratory workforce

**DOI:** 10.4102/ajlm.v15i1.2958

**Published:** 2026-07-22

**Authors:** Elias Chipofya

**Affiliations:** 1 Malawi Association of Medical Laboratory Scientists, Lilongwe, Malawi

## Introduction

Medical laboratories are the unseen foundation of healthcare, but they are often undermined by inadequate resources, a critical reality across Malawi and sub-Saharan Africa.^1^ This deficit is alarming because laboratory quality guides most clinical decisions, making its reliability paramount for patient outcomes and safety. Improving the quality of laboratory services requires a critical shift from mere test volume to prioritising quality, clinical impact, and utility – termed value-based.^2^ Values-based laboratory medicine requires laboratory professionals to demonstrate the clinical value of their discipline by emphasising test appropriateness and sustainability, and by integrating advanced technologies like artificial intelligence throughout the total testing process.^3^ Achieving this transition necessitates strong leadership that extends beyond local service directors to engage national and international specialist organisations.^4^ This collective leadership holds the core responsibility for ensuring that laboratory services are used optimally for the benefit of both the patient and the entire healthcare system.

Malawi’s healthcare is delivered through three primary provider types: (1) the free public system; (2) private for-profit facilities; and (3) private not-for-profit facilities. The public system is the largest, managed by district councils and ministries. Crucially, the private not-for-profit sector is supported significantly by the Christian Health Association of Malawi, a mission hospital network delivering nearly 30% of all healthcare services. Private for-profit facilities are wholly privately owned. The entire system relies on four interconnected service levels for streamlined patient referral. Services begin at the community level and feed into primary care (health centres and/or community hospitals). Referrals then ascend through secondary care (district hospitals) to the highest level, tertiary care, handled by five central hospitals in major cities.^5^

Despite this infrastructure, Malawi is gripped by a severe dual public health crisis: high human immunodeficiency virus/acquired immunodeficiency syndrome (HIV/AIDS) prevalence alongside rapidly increasing non-communicable diseases, creating complex comorbidity that strains healthcare provision severely.^6^ Accurate and timely laboratory diagnostics are essential to address these integrated challenges effectively, and a highly skilled and supported medical laboratory workforce is crucial to the delivery of the necessary quality of service. These professionals generate the evidence needed to inform policy, guide interventions, and track progress toward Sustainable Development Goal 3 (SDG 3). Malawi’s success in achieving the 2030 Sustainable Development Agenda hinges on this reliable health data. The laboratory can also support other SDGs, such as clean water (SDG 6) and responsible consumption (SDG 12), through sustainable practices.^7^

Policy deficits in Malawi are the core issue, directly impeding the development of consultant-level expertise (senior professionals who can design and direct clinical and/or scientific laboratory services^8^) and driving widespread dissatisfaction among healthcare workers. This affects laboratory medicine professionals with advanced qualifications (Master of Science [MSc] or Doctor of Philosophy [PhD]) particularly, fuelling a damaging cycle of ‘brain drain’, ‘brain waste’, and professional devaluation.^9,10^ This high risk of internal attrition of the specialist workforce is driven by specific systemic failures, including extremely low pay (the lowest satisfaction dimension), a lack of career advancement owing to delayed promotion and development and, critically, a lack of recognition of advanced academic qualifications, all of which are compounded by inadequate infrastructure and poor working conditions.^1,11,12^ This opinion paper aims to analyse these deficits and to propose evidence-based retention and motivation strategies for highly skilled laboratory medicine personnel, acknowledging their vital specialist role in accurate diagnosis, disease surveillance, and national health security. It sets out a thematic review of national and multi-country studies that delineate these policy-driven systemic factors. The thematic selection and interpretation of the cited literature were guided by the core objective of the opinion paper: linking systemic policy failures to the specific consequences of brain drain and brain waste in Malawi’s laboratory sector. Cited articles were chosen based on their direct relevance in defining policy-driven systemic factors (e.g. salary, promotion delay, recognition) identified as primary drivers of dissatisfaction in the Malawian health workforce, alongside studies that validate the vital role of laboratory medicine professionals in public health security. The interpretation synthesises these findings to demonstrate a clear causal pathway from political and structural deficits to the underutilisation and attrition of highly skilled personnel, thereby justifying the policy recommendations presented.

## Understanding the ‘brain drain’ and ‘brain waste’: Causes of migration and demotivation

The departure and underutilisation of Malawi’s medical laboratory professionals are rooted in complex systemic issues. While the Malawi Ministry of Health (MoH) has long struggled with attracting, motivating, and retaining health professionals^13^ (a challenge mirrored in Ghana),^14^ a recent 2025 study indicates that the situation is deteriorating actively owing to the unintended consequences of the structural reforms of the MoH and specific barriers for specialised staff.^5,12^ The poor implementation of functional reviews (a structured assessment of an organisation’s operations to evaluate effectiveness and efficiency) has been found to exacerbate occupational stress among health workers.^5^ This destabilisation is linked to the chronic dissatisfaction of specialised workers, who continue to cite low salary/benefits and limited career advancement as the lowest satisfaction dimensions, fuelling their high intention to leave government service.^12^

A recent World Health Organization survey exposed Malawi’s critical health workforce crisis. The country’s density of physicians, nurses, and midwives is less than 0.5 per 1000 population, placing it drastically below the 4.45 per 1000 SDG threshold for Universal Health Coverage.^15^ This shortage is widespread; for instance, the nation had only 542 laboratory personnel in 2018. However, the current catastrophic state of Malawi’s medical laboratories is due primarily to a critical leadership deficit. The Medical Council of Malawi, the statutory body responsible for regulating and registering all allied health professionals, including laboratory medicine experts, reports an extreme scarcity of top-tier laboratory medicine expertise. As of 08 October 2025, only seven registered Biomedical Scientists (the nation’s most senior laboratory experts [MSc or PhD holders]) are available to oversee services for Malawi’s 22.2 million people. This crisis is compounded by high attrition: since registration began, only 18 professionals have ever joined the Register, and for the 2025/26 financial year, 11 failed to renew their certificate (a 39% renewal rate), indicating a strong likelihood of their emigration. This deficit is staggering: the European standard recommends 10 senior specialists per million people,^16^ while a sophisticated need-adjusted demand model indicates that the true national requirement for Malawi is closer to 333 laboratory medicine specialists.^17^ In short, the country is missing an incredible 97.9% of the specialised laboratory medicine leadership required, unequivocally threatening the safety and accuracy of patient diagnoses nationwide. Given that modern laboratory medicine specialists are crucial for facilitating clinical liaison, integrating automation, managing quality, and synthesising complex diagnostic data to inform patient management, this leadership void severely compromises patient-centred care and clinical decision-making across the country.^3^

### Inadequate remuneration and limited career progression

The principal driver of attrition for Malawian medical laboratory professionals is the uncompetitive salary structure of the public sector. This financial inadequacy is demonstrated by the critically low overall job satisfaction levels of the health workforce in primary care settings (8.1%), with satisfaction regarding remuneration and recognition dismal at just 9.8%.^11^ This profound dissatisfaction, which prevents professionals from meeting basic needs and securing their future, is compounded by government budget constraints that impose wage freezes and restrict new graduate hiring, resulting in unemployment or underemployment.^5,9^ This vulnerability is immediately exploited by non-governmental organisations in Malawi, which attract public sector staff with higher salaries, thus fuelling broader healthcare professional migration from low- and middle-income countries.^12,14^

Financial strain is worsened severely by unfair internal salary structures rooted in the systemic undervaluation of laboratory medicine. For instance, 5-year Honours graduates in Medical Laboratory Science (BSc Hon MLS) are recruited at a lower grade (I/M6) than graduates of the comparable 5-year Bachelor of Biomedical Engineering Honours (BBmE Hons) programme (H/M5). This discrepancy is not an administrative error; it signifies a profound lack of recognition.^1^ The lower grading of the MLS cadre stems from the historically lower visibility of the profession, despite essential diagnostic work being pivotal for prevention, diagnosis, and treatment.^3,18^ This contrasts sharply with the higher grade for BBmE, which reflects a strategic premium paid to technical experts to address equipment scarcity.^19^

Compounding the challenges in the Malawian health sector is the rigid Human Resources Management system, which is dictated centrally by the *Public Service Act* (Chapter 1:03). This structure enforces a post-centred bureaucracy, meaning that appointments are tied strictly to filling established, vacant posts.^20^ This creates a profound policy paradox: while high-level bureaucrats leverage this rigidity strategically as everyday acts of resistance^21^ to assert domestic power and curtail the influence of external development partners, the same framework dismantles clear, attractive career paths for domestic professionals.^1^ This structure, therefore, prioritises national policy sovereignty at the expense of internal professional development.

This external pressure exacerbates internal flaws: promotions frequently favour tenure over specialised education, leading directly to the phenomenon of ‘brain waste’. These Human Resources Management failures are a chronic issue, having been identified as a key challenge to health professional retention since at least 2006.^22^ They continue to drive professional dissatisfaction, manifesting as an identity crisis among key cadres like clinical officers who shoulder doctor-level responsibilities without commensurate recognition,^5^ and contribute to broader health policy and planning challenges.^23^ The consequences for talent retention remain significant, exemplified by medical doctors with postgraduate qualifications who returned expecting MoH promotion but were compelled to resign or to spend months negotiating their posting when recognition failed to materialise owing to bureaucratic barriers.^22^ Indeed, promotions are based solely on work experience, meaning that an MSc or PhD holder might report to a BSc holder who was promoted earlier. This deficit in defined, compensated career routes subsequently forces skilled professionals, such as laboratorians, to deviate to non-laboratory disciplines, like public health, in search of upward career mobility.^12,18^

Furthermore, systemic reform efforts designed to streamline the workforce have struggled to operationalise strategy. While the MoH launched the *Health Sector Strategic Plan III (HSSP III) 2023–2030* to unify the fragmented sector, this shift is hampered significantly by incomplete operational plans and misaligned decision-making systems, normalising the status quo and impeding the necessary long-term reform.^23^ Even functional reviews, which were intended to achieve organisational efficiency, have introduced severe unintended human costs. For example, the 2014 restructuring of the MoH triggered the reclassification of some clinical officers who, owing to an acute shortage of medical doctors in Malawi, were already tasked with performing duties that fall conventionally in the purview of the former. This reclassification was perceived widely as a demotion, triggering a profound identity crisis and severely exacerbating occupational stress amongst staff.^5^ This pervasive professional disenchantment is a significant barrier to effective interprofessional collaborative practice. Ultimately, this nexus of uncompetitive pay, structural devaluation, bureaucratic rigidity, political interference, and failed reform inhibits laboratory medicine professional retention critically.^1^

### Chronic operational challenges and the imperative for advanced local analytical capacity and Medical Laboratory Science leadership

Malawian medical laboratories face significant hurdles, compromising service quality and demotivating professionals. These challenges, including inadequate infrastructure, outdated equipment, and chronic shortages of essential reagents, are particularly concerning given the rising demand for quality laboratory services.^1,24^ For instance, the new National Cancer Centre at Kamuzu Central Hospital, which opened on 02 June 2025, will rely heavily on high-quality local laboratory support for its success. Indeed, the need for advanced local capabilities is critical. The absence of real-time drug monitoring for crucial chemotherapeutic agents^25^ and the inherent complexities of estimating drug exposure accurately^26^ demand highly adaptable laboratory practices. This lack of advanced local analytical capacity is not new; past research highlights its importance consistently. Studies on drug metabolism in malnourished children^27^ and toxic metals in pregnant women^28^ both required analyses to be undertaken overseas owing to a lack of advanced equipment locally. These issues underscore the critical need for robust local infrastructure, as poor working conditions and inadequate resources are significant ‘push factors’ for professionals to leave.^14^

The indisputable value of laboratory medicine is undermined by its low visibility.^1,3^ Globally, the discipline has evolved into a dominant, patient-centred role, influencing clinical decisions strongly for early diagnosis and targeted therapy.^2^ This modern role, essential for national initiatives like the National Cancer Centre, demands a shift towards value-based laboratory medicine, requiring the discipline to provide evidence actively of its clinical impact.^2,3^

This necessitates highly skilled consultant-type leadership (holding an MSc or PhD). Laboratory medicine leaders need competencies beyond technical compliance, including strategic decision-making and financial management.^4^ They must also manage the pre-analytical and post-analytical phases of testing expertly, where most errors and value addition occur.^29^ This complexity is heightened by advanced diagnostic technologies such as high-resolution mass spectrometry, now the gold standard for therapeutic drug monitoring and other key applications.^3^ Until the need for specialised, recognised, and properly compensated leadership is met, the full potential of laboratory medicine to drive healthcare innovation will remain unrealised.

Compounding these issues are severe staff shortages, unclear job descriptions, and insufficient supervision, all contributing significantly to burnout. This is exacerbated by biosafety and occupational health concerns, often worsened by inadequate protective equipment, creating hazardous work environments. This persistent devaluation of the workforce fosters what is commonly termed ‘brain waste’, severely reducing morale and prompting professionals to seek more respected and secure environments.

The core attrition drivers identified nearly a decade ago by Chipeta at Kamuzu Central Hospital^30^ (poor pay, high workload, limited career paths, and inadequate resources) remain critically relevant today, particularly for specialised groups like medical laboratory scientists (MLS). This enduring problem is quantified starkly by current data. Muhula et al. highlight an exceptionally low (9.8%) satisfaction rate with remuneration among health workers in Malawi,^11^ resulting in a median facility-service duration of a mere 3 years. This dissatisfaction with low pay and non-existent career advancement is mirrored across specialised mid-level cadres, where studies show that over 60% of clinicians have considered leaving their public sector posts.^12^ Furthermore, despite national training institutions producing thousands of graduates, budgetary constraints prevent the public sector from creating the necessary posts, leading to high rates of unemployed or underemployed MLS staff.^9^ This paradoxical shortage acts as a powerful push factor for both brain drain and brain waste.

Furthermore, MLS-specific analyses confirm that the cadre suffers from poorly equipped facilities, frequent shortages of supplies and reagents, and a lack of formal professional development.^1^ The resulting staff shortages, low morale, and operational deficiencies consistently compromise the ability of the healthcare system to provide quality diagnostic services during public health emergencies. In 2020, this systemic failure was extensively demonstrated during the coronavirus disease 2019 pandemic, where testing efforts were crippled by inadequate capacity, insufficient funding, and a lack of qualified laboratory human resources despite the existence of a pool of unemployed experts.^31^ The crisis continued to manifest during the 2022–2023 cholera outbreak.^9^

Despite these systemic impediments to technical and leadership development, the public health laboratory system demonstrates remarkable resilience and specialised capacity in Malawi: this is evidenced by its rapid coronavirus disease 2019 testing network expansion^31^; successful establishment of rigorous clinical research cohorts, specifically a prospective observational cohort of hospitalised coronavirus disease 2019 patients over four epidemic waves^32^; ISO 15189:2012 accreditation for nine out of 10 HIV molecular labs, an achievement facilitated by a hybrid mentorship model within 2 years^33^; and successful in-country genomic sequencing during the 2022–2023 cholera outbreak.^34^

## Strategies for retaining and motivating Malawi’s medical laboratory workforce

Addressing Malawi’s laboratory workforce ‘brain drain’ and ‘brain waste’ requires valuing and supporting these critical professionals using a range of strategies.

### Foundational governance: Structural and legislative reform

Malawi’s health sector presents a complex landscape, marked by significant strengths in its foundational structure and the intrinsic motivation of its dedicated workforce, but simultaneously constrained by pervasive systemic challenges related to human resources, governance, and the execution of policy.^1,5,9,11,24^

#### Action required

Decentralise Human Resources Management by implementing policy and/or legislative reforms to grant local authorities (comprising four cities, 28 district councils, two municipal councils, and one town council) and the MoH autonomy over specialist promotions and/or compensation. This ensures that advanced degrees (MSc or PhD) lead to competitive seniority and recognition, retaining experts needed for technological leadership and complex data interpretation, and sustainably countering brain drain.^3^

#### Rationale

The current centralisation of Human Resources functions acts as the structural block that prevents local and line management from appointing and rewarding the most qualified staff. Granting this joint autonomy to the 35 single-tier local authorities and the MoH is the foundational, cost-effective reform required to address political interference and enable the person-centric system at the point of service delivery.

### Strategic financing: Resource mobilisation and phased pay

#### Action required

Implement strategic resource mobilisation and phased salary increases.

#### Rationale

To overcome the affordability constraint, the government must adopt a targeted financing strategy. This involves engaging donors with a costed Human Resources for Health plan and prioritising salary increases for the most critically deficient, highly qualified cadres (MSc or PhD) over a defined period. This will ensure that limited funds generate maximum return and secure external financing. This approach is essential because it addresses brain waste directly by ensuring that advanced training leads immediately to enhanced status, responsibility, and compensation. This necessity is supported by the academic recommendation that PhD-level training must be associated with defined career paths adequately compensated to minimise loss of laboratory professionals to other disciplines and align with the general call for more investment in laboratory medicine training and systems.^18^

### Professional environment, working conditions, and governance

#### Action required

Implement and enforce rigorously comprehensive quality management systems, aiming for international accreditation (e.g., ISO 15189), backed by targeted government and donor investment in modernising laboratory infrastructure and ensuring optimal staffing.

#### Rationale

This holistic approach tackles the work environment and governance. Quality management system is a cost-effective professional driver, but it cannot be achieved without adequate resources and staffing. Investment must be prioritised for modernising infrastructure, including establishing advanced facilities for complex analyses (like drug monitoring using high-resolution mass spectrometry, currently outsourced). This does not only address the operational needs of the profession but is essential for improving quality local patient care by reducing outsourcing and enabling timely, accurate, complex diagnostics. Furthermore, mandating active MLS participation in policy and regulatory bodies will ensure that investment decisions align with both operational needs and patient safety goals.^1^

## Conclusion

Malawi’s national public health is threatened gravely by the ‘brain drain’ and ‘brain waste’ of medical laboratory professionals. This stems from inadequate pay, limited career growth, poor working conditions, and systemic non-recognition of advanced qualifications, pushing skilled individuals abroad or leading to underutilisation of their abilities. Addressing this requires comprehensive policy reforms. By ensuring competitive and fair pay, clear career paths that recognise advanced degrees formally, improved working conditions, and genuine empowerment of specialist professionals, Malawi can build a robust, motivated, and highly skilled medical laboratory workforce. This investment in the unseen foundation of healthcare is vital for accurate diagnoses, effective treatments, resilient public health surveillance, and ultimately, achieving its Sustainable Development Goals.
